# Efficacy of the Enhanced Recovery After Surgery program for thoracic surgery in a developing country

**DOI:** 10.1007/s12055-023-01518-3

**Published:** 2023-05-16

**Authors:** Sira Laohathai, Zarina Sadad, Kanok Suvarnakich, Chompunoot Pathonsamit, Apichat Tantraworasin

**Affiliations:** 1 Cardiothoracic Surgery Unit, Department of Surgery, Faculty of Medicine, Vajira Hospital, Navamindradhiraj University, Bangkok, Thailand; 2 Department of Anaesthesia, Faculty of Medicine, Vajira Hospital, Navamindradhiraj University, Bangkok, Thailand; 3 Clinical Surgical Research Centre, Faculty of Medicine, Chiang Mai University, 110 Intrawaroros Road, Chiang Mai, Thailand; 4 Thoracic Surgery Unit, Department of Surgery, Faculty of Medicine, Chiang Mai University, Chiang Mai, Thailand

**Keywords:** Enhanced Recovery after Surgery, Open thoracotomy, Video-assisted thoracic surgery, ERAS protocol

## Abstract

**Purpose:**

Enhanced Recovery After Surgery (ERAS) is a strategy used to improve perioperative outcomes and reduce complications. However, data on the efficacy of ERAS in thoracic surgery in developing countries are limited. The current study aimed to validate the benefits of ERAS among patients at a single institution.

**Methods:**

This was a retrospective study of patients who underwent pulmonary resection at Vajira Hospital, Bangkok, Thailand, between 2016 and 2020. To compare outcomes, patients were divided into the pre-ERAS group (2016–2018) and the post-ERAS group (2019–2020) using propensity score matching (1:2) with the year 2019 as the cutoff for introducing ERAS protocols at our institution.

**Results:**

In total, 321 patients were included in the analysis (pre-ERAS group, *n* = 74; post-ERAS group, *n* = 247). After propensity score matching, 56 and 112 patients were classified under the pre- and post-ERAS groups, respectively. The post-ERAS group had significantly lower pain scores than the pre-ERAS group on postoperative days 1, 2, and 3, and a lower volume of intraoperative blood loss. In the multivariable analysis, the post-ERAS group had a shorter chest tube duration (mean difference = −1.62 days, 95% confidence interval = −2.65 to −0.31) and length of hospital stay (mean difference = −2.40 days, 95% confidence interval = −4.45 to −0.65) than the pre-ERAS group.

**Conclusion:**

The use of ERAS guidelines in pulmonary resection is beneficial. Although no significant differences were observed in postoperative complication rate, intensive care unit stay, and additional cost burden between the two groups, patients in the post-ERAS group had a shorter postoperative chest tube duration, shorter hospital stays, shorter operative time, lower postoperative pain score, and lower volume of intraoperative blood loss.

## Introduction

Enhanced Recovery After Surgery (ERAS) is a program for colorectal surgery that was first introduced by Kehlet in 1990 [1, 2]. It aims to decrease the incidence of postoperative complications, shorten the length of hospital stay, and reduce hospitalization costs. Currently, the benefits of ERAS for thoracic surgery are still debated. Previous studies have shown that it is beneficial in terms of surgical outcomes. However, some had contrasting results [3–5]. Data on ERAS in thoracic surgery in Thailand are limited. Therefore, the current retrospective study evaluated the benefits of ERAS among patients at a single institution.

## Materials and methods

This retrospective study included patients who underwent elective pulmonary resection at the Cardiothoracic Surgery Unit, Department of Surgery, Faculty of Medicine, Vajira Hospital, Navamindradhiraj University, Bangkok, Thailand, between 2016 and 2020. Emergency cases were excluded. In 2019, the ERAS protocols were introduced in patients undergoing surgery at our institution. Data on patients who underwent elective pulmonary resection between 2016 and 2020 were retrospectively extracted from medical records. Participants were divided into the pre-ERAS group (*n* = 74) and the post-ERAS group (*n* = 247) based on the introduction of the ERAS protocol. The groups were compared for intraoperative and postoperative outcomes, including duration of chest tube drainage, hospital stay, volume of intraoperative blood loss, postoperative complications (e.g., arrhythmia and pneumonia), and postoperative mortality rates. The primary outcome was the length of hospital stay, and the secondary outcome was chest tube duration.

### Pre-ERAS protocol

The patients were not instructed to quit smoking and were not provided pre-analgesia medications preoperatively. Further, during surgery, the patients did not receive local anesthesia or intercostal nerve block. Operative care and postoperative care were routinely provided based on the surgeon’s preference.

### ERAS protocol

Table 1 shows the protocols in the ERAS program. These protocols were adapted from the guidelines for enhanced recovery after lung surgery recommended by the Enhanced Recovery After Surgery Society and the European Society of Thoracic Surgeons [6].

**Table 1:** Protocol for patients undergoing thoracic surgery

**Preoperative issues**	**Action**
Anemia	Diagnosis and correction (Hb > 11 in women, Hb > 12 in men)
Nutrition	Nutrition screening and support
Smoking cessation	Advice on smoking cessation
Poor preoperative fitness/lack of physical activity	Physical exercise training
Rehabilitation	Teach breathing exercises before surgery
Fasting requirement	A minimal fasting period of 6 h prior to surgery
**Intraoperative issues**	**Action**
Antibiotics	Routine administration of prophylaxis antibiotics
Pain control	Preemptive analgesia
Multimodal analgesia (induction with propofol and maintenance with remifentanil. Regional anesthesia included a paravertebral intercostal blockade with bupivacaine	
Fluids	Very restrictive or avoided liberal fluid regimens in favour of euvolemia
Ventilation	Provide lung-protective strategies during one-lung ventilation
Temperature	Prevent hypothermia
Tracheal extubation	Attempt to extubate in all cases
Surgical access	Minimal invasive surgery with one-lung ventilation
Chest drain	Single chest drain
Central venous catheter and urinary catheter	Use only when necessary
**Postoperative issues**	**Action**
Chest drain	Early removal of drains between 12 to 24 h after the surgical intervention if air leakage ceased, fully expanded lung
Chest X-ray	Evaluated daily till the chest drain was removed
Laboratory tests	Laboratory tests were required once the patient showed any abnormality on physical examination when compared to the preoperative anaesthetic and cardiovascular evaluation
Mobilization	Aggressive early mobilization within 24 h of surgery
Oral intake	Early oral intake
Caregiver counselling	Providing the postoperative care guidance to patients and their family member
Hospital discharge	Make an appointment to return to the thoracic surgery clinic within 14 days to continue the postoperative follow-up

### Preoperative preparation

All patients were assessed for preoperative functionality and provided with assistance in smoking cessation at least 2 weeks before surgery. Patients diagnosed with anemia (hemoglobin < 11 g/dL in women and < 12 g/dL in men) were treated.

### Intraoperative management

Preoperative analgesic medication (paracetamol and nonsteroidal anti-inflammatory drugs) was routinely administered on the night before surgery. Patients were required to fast for at least 6 h before surgery and were instructed not to drink fluids for at least 2 h prior to surgery. Antibiotic prophylaxis was administered 60 min before surgery. Central venous access and urinary catheterization were considered for patients at high risk of hemodynamic instability, such as those with cardiovascular disease and extended surgery (pneumonectomy or sleeve lobectomy). In addition to intercostal nerve block administered between the third and eighth intercostal spaces before the end of surgery, local anesthesia was routinely injected at the surgical site before and after surgery.

### Surgical protocol

All procedures were performed under general anesthesia using the lung isolation technique with a double-lumen endotracheal tube or bronchial blocker. Surgery was performed by board-certified cardiovascular thoracic surgeons. The use of video-assisted thoracic surgery (VATS) or open thoracotomy (OT) was based on the surgeon’s preference.

In OT, the standard posterolateral thoracotomy technique was routinely used. In VATS, the number of ports was based on the technique selected by the surgeon. However, uniportal VATS with a 3–4-cm incision at the fifth intercostal space on the anterior axillary line for utility ports was routinely performed.

After surgery, a chest tube (24 or 28 Fr) was routinely inserted at the incision site under endoscopic view and was placed posterosuperior in the pleural cavity. The patient was extubated immediately after the surgery if possible. Chest radiography was performed within 8–12 h postoperatively.

### Postoperative management

To enhance postoperative recovery, we encouraged patient independence as early as possible. Our rehabilitation team could facilitate breathing and mobility exercises and provide tri-flow to patients during postoperative care. Multimodal painkillers, which combined paracetamol and nonsteroidal anti-inflammatory drugs, were prescribed to all patients except for those with underlying kidney disease, who received opioids and anti-neuropathic medications. Paravertebral or epidural blocks were not routinely performed.

A single intercostal chest drain was placed and connected to a wall suction system. If complete lung expansion was observed, the thoracic wall suction was removed. The intercostal chest drain was removed if there was less than 300 mL of non-hemorrhagic pleural fluid and no air leakage. Treatment with supplementary oxygen was discontinued if oxygen saturation on room air reached more than 92%.

### Discharge

Patients were discharged a day after their chest drain was removed, and they were followed up at our outpatient clinic after 2 weeks.

### Statistical analysis

Categorical variables were presented as frequencies and percentages and continuous variables as medians and interquartile ranges (P_25_–P_75_). A propensity score matching analysis (1:2) was performed to minimize selection bias in the pre- and post-ERAS groups. The logistic regression model was used to calculate propensity scores, which can be used to evaluate confounding by indication and/or baseline covariates between the pre- and post-ERAS groups. The variables included in the propensity score matching model were age, sex, diagnosis, laterality, and type of operation. A standardized mean difference between groups was determined for all covariates. A standardized mean difference of < 0.2 indicated non-significant difference. The chi-square tests were used to compare categorical variables and differences between the two groups for dichotomous data. The Student’s *t*-test was utilized to compare continuous data with normal distribution, and the Mann-Whitney *U* test was applied for continuous data with skewed distribution. Multivariable regression analyses were performed to assess the association between two groups and significant outcomes. We also analyzed the interaction between ERAS protocol and surgical approaches (VATS vs. OT) to identify the benefit of using ERAS protocol regardless of surgical approaches. A *p*-value of < 0.05 was considered statistically significant. All statistical analyses were performed using STATA v. 16.0 software (StataCorp, College Station, TX, USA).

## Results

In total, 321 patients underwent pulmonary resection at Vajira Hospital between 2016 and 2020. Among them, 74 underwent surgery before the introduction of ERAS and 247 under ERAS. Before propensity matching, the pre-ERAS group was slightly older than the post-ERAS group (66.9 vs. 60 years; *p* < 0.001). There were no significant differences between the two groups in terms of sex, smoking status, and underlying conditions. The proportion of patients who underwent lobectomy in the pre-ERAS group was higher than that of patients in the post-ERAS group (54.1% vs. 36.1%; *p* = 0.002). Table 2 presents the demographic and clinical characteristics of the patients.

**Table 2:** Patient’s demographic compares between pre- and post-ERAS before propensity match score

Basic descriptive	Before propensity score matching				After propensity score matching			
	(Full patient cohort)				(Propensity score matched patient cohort)			
	Pre-ERAS (*n* = 74)	Post-ERAS (*n* = 247)	*p*-value	SMD	Pre-ERAS (*n* = 56)	Post-ERAS (*n* = 112)	*p*-value	SMD
**Age; median (P**_**25**_**–P**_**75**_**)**	66.95 (60.3**–**71)	60 (38.9**–**68.1)	< 0.001*	0.586	66.95 (60.2**–**70.95)	65 (9**–**73)	0.882	0.040
**Gender; *n* (%)**			0.548	0.079			0.827	0.036
Male	36 (48.6%)	130 (52.6%)			28 (50%)	58 (51.8%)		
Female	38 (51.4%)	117 (47.4%)			28 (50%)	54 (48.2%)		
**Smoking status; *n* (%)**			0.414	0.111			0.669	0.070
Non-smoker	63 (85.1%)	200 (81%)			47 (83.9%)	91 (81.3%)		
Smoker	11 (14.9%)	47 (19%)			9 (16.1%)	21 (18.8%)		
**Underlying disease; *n* (yes%)**								
Diabetes	15 (20.3%)	45 (18.2%)	0.691	0.052	10 (17.9%)	26 (23.2%)	0.425	−0.132
Hypertension	27 (36.5%)	82 (33.2%)	0.600	0.099	19 (33.9%)	53 (47.3%)	0.098	−0.273
Dyslipidemia	19 (25.7%)	53 (21.5%)	0.445	0.069	13 (23.2%)	29 (25.9%)	0.705	−0.062
Cardiovascular disease	5 (6.8%)	14 (5.7%)	0.728	0.045	5 (8.9%)	10 (8.9%)	1.000	0.000
**Operation type; *n* (%)**			0.002*	0.467			0.683	0.141
Wedge	32 (43.2%)	158 (64%)			25 (44.6%)	57 (50.9%)		
Segmentectomy	2 (2.7%)	11 (4.5%)			1 (1.8%)	1 (0.9%)		
Lobectomy	40 (54.1%)	78 (31.6%)			30 (53.6%)	54 (48.2%)		
**Diagnosis; *n* (%)**			< 0.001*	0.811			1.000	0.000
Benign	1 (1.4%)	69 (27.9%)			1 (1.8%)	2 (1.8%)		
Cancer	73 (98.6%)	178 (72.1)			55 (98.2%)	110 (98.2%)		
**Left side surgery**	21 (28.4%)	104 (42.1%)	0.034*	0.290	18 (32.1%)	37 (33%)	0.907	0.019

*SMD* standardized mean difference

Intraoperatively, the post-ERAS group was more likely to undergo VATS than the pre-ERAS group (93.5% vs. 16.2%; *p* < 0.001). There were no significant differences in the incidence of blood transfusions, arrhythmia, or 30-day mortality between the two groups. The post-ERAS group had a shorter operative duration and lower volume of intraoperative blood loss than the pre-ERAS group (60 vs. 180 min, *p* < 0.001 and 20 vs. 100 mL, *p* < 0.001). The post-ERAS group had a shorter chest tube duration (3 vs. 4 days, *p* < 0.001) and length of hospital stay (6 vs. 11 days, *p* < 0.001) than the pre-ERAS group. The post-ERAS group had a higher incidence of postoperative complication than the pre-ERAS group. The proportion of patients who were admitted to the intensive care unit postoperatively in the post-ERAS group was higher than that of patients in the pre-ERAS group (5.4% vs. 15.4%; *p* < 0.026). Table 3 presents all data in detail.

**Table 3:** Patient’s demographic compares between pre- and post-ERAS before propensity match score

Variables	Before propensity score matching				After propensity score matching			
	(Full patient cohort)				(Propensity score matched patient cohort)			
	Pre-ERAS (*n* = 74)	Post-ERAS (*n* = 247)	*p*-value	SMD	Pre-ERAS (*n* = 56)	Post-ERAS (*n* = 112)	*p*-value	SMD
Blood transfusion; *n* (no%)	4 (5.4%)	4 (1.6%)	0.067	0.206	4 (7.1%)	3 (2.7%)	0.172	0.207
Perioperative complication; *n* (%)			0.740	0.128			0.478	0.134
Arrhythmia	0 (0%)	2 (0.8%)			0 (0%)	1 (0.9%)		
Approaches; *n* (%)			< 0.001*	2.467			< 0.001*	2.444
VATS	12 (16.2%)	231 (93.5%)			10 (17.9%)	104 (92.9%)		
Open thoracotomy	62 (83.8%)	16 (6.5%)			46 (82.1%)	8 (7.1%)		
Postoperative complication; *n* (%)								
Pneumonia	1 (1.4%)	3 (1.2%)	0.926	0.012	1 (1.8%)	2 (1.8%)	1.000	0.000
Hoarseness	0 (0%)	4 (1.6%)	0.271	0.181	0 (0%)	2 (1.8%)	0.314	0.191
Prolong air leak	0 (0%)	8 (3.2%)	0.206	−0.258	0 (0%)	3 (2.7%)	0.552	−0.234
Atrial fibrillation	1 (1.4%)	1 (0.4%)	0.408	0.101	1 (1.8%)	0 (0%)	0.333	0.189
Acute kidney injury	0 (0%)	2 (0.8%)	1.000	−0.128	0 (0%)	1 (0.9%)	1.000	−0.134
Other	3 (4.1%)	9 (3.6%)	1.000	0.021	1 (1.8%)	5 (4.5%)	0.603	−0.154
Mortality; *n* (%)	0 (0%)	3 (1.2%)	0.400	0.223	0 (0%)	1 (0.9%)	0.603	0.191
Immediate extubation; *n* (%)	70 (94.6%)	235 (95.1%)	0.850	0.025	52 (92.9%)	107 (95.5%)	0.483	0.115
Postoperative ICU stay; *n* (%)	4 (5.4%)	38 (15.4%)	0.026*	0.387	3 (5.4%)	23 (20.5%)	0.010*	0.464
Chest tube duration stay; median (P_25_–P_75_)	4 (2–8)	3 (1–4)	< 0.001*	0.445	4 (2, 5)	3 (2, 4)	0.017*	0.359
Hospital stay; median (P_25_–P_75_)	7 (6–11)	4 (3–6)	< 0.001*	0.450	7 (6, 11)	5 (3, 7)	< 0.001*	0.454
Operative time; median (P_25_–P_75_)	180 (120–2400)	60 (40–120)	< 0.001*	1.208	180 (125–240)	70 (45–150)	< 0.001*	1.053
Estimate blood loss; median (P_25_–P_75_)	100 (42.5–150)	20 (10–50)	< 0.001*	0.238	100 (50–150)	20 (10–98.8)	< 0.001*	0.239
Pain score (0–10)								
Day 1; median (P_25_–P_75_)	7 (7–8)	3 (2–5)	< 0.001*	1.847	7 (7–8)	3 (1.5–5)	< 0.001*	1.836
Day 2; median (P_25_–P_75_)	7 (4.8–8)	0 (0–2)	< 0.001*	2.240	7 (5–8)	0 (0–2)	< 0.001*	2.181
Day 3; median (P_25_–P_75_)	3.5 (1–7)	0 (0–0)	< 0.001*	1.463	3.5 (1–7)	0 (0–0)	< 0.001*	1.425

*SMD* standardized mean difference

### Propensity score matching

The post-ERAS group had a shorter operative time and lower volume of intraoperative blood loss than the pre-ERAS group (70 vs. 180 min, *p* < 0.001 and 20 vs. 100 mL, *p* < 0.001, respectively). The post-ERAS group had a shorter intubation duration and length of hospital stay than the pre-ERAS group (4 vs. 4 days, *p* = 0.017 and 5 vs. 7 days, *p* < 0.001, respectively). Compared with the pre-ERAS group, the post-ERAS group had lower pain scores on postoperative days 1, 3, and 3 after surgery (3 vs. 7, *p* < 0.001; 0 vs. 7, *p* < 0.001; and 0 vs. 3.5, *p* < 0.001, respectively). There were no significant differences in terms of rates of blood transfusion, arrhythmia, immediate extubation, and 30-day mortality. Table 3 shows the variables between the two groups.

After propensity score matching, the surgical approach (VATS vs. OT) was still not balanced because VATS was introduced at our institution at the same time as the development of the ERAS protocol. Therefore, the effect of minimally invasive procedure might inhibit the effect of the ERAS protocol. Further, multivariable regression analysis adjusted for confounding factors that might be associated with outcomes was conducted. Additionally, we explored the benefits of the ERAS protocol regardless of surgical approach by testing for interaction between using ERAS protocol and surgical approaches, as presented in Table 4. In the first row of each outcome variable (post-ERAS vs. pre-ERAS), the ERAS protocol was still advantageous as it was associated with a shorter chest tube duration and length of hospital stay in both OT and VATS. For the remaining rows of each outcome variable, which represent the interaction testing between using ERAS protocol and surgical approaches, post-ERAS group with OT had a shorter chest tube duration and a shorter length of hospital stay when compared to the pre-ERAS with OT. This result demonstrates that even though OT was performed in the post-ERAS group, the outcomes were still superior to those in the pre-ERAS group with OT.

**Table 4:** The interaction between ERAS protocol and surgical approaches in outcome variables

Outcome variables	Coefficient	95% CI	*p*-value*
Chest tube duration time (day)			
Post-ERAS versus pre-ERAS	−1.62	(−2.65) to (−0.31)	0.011
Pre-ERAS and open thoracotomy	Reference		
Pre-ERAS and VATS	−3.36	(−3.74) to (−2.97)	<0.001
Post-ERAS and open thoracotomy	−2.06	(−2.51) to (−1.61)	< 0.001
Post-ERAS and VATS	−2.58	(−2.66) to (−2.50)	< 0.001
Hospital stays (day)			
Post-ERAS versus pre-ERAS	−2.40	(−4.45) to (−0.65)	0.005
Pre-ERAS and open thoracotomy	Reference		
Pre-ERAS and VATS	−2.44	(−2.67) to (−2.20)	< 0.001
Post-ERAS and open thoracotomy	−1.15	(−1.40) to (−0.90)	< 0.001
Post-ERAS and VATS	−2.05	(−2.11) to (−1.99)	< 0.001

Analyzed by generalized linear regression model adjusted by age, gender, postoperative complications, surgeons, and year at surgery

## Discussion

The first ERAS guidelines for colorectal surgery were published in 2005 [7]. Thereafter, several guidelines for various medical fields, including orthopedics, urology, and thoracic surgery, have been established [8, 9]. However, implementing these programs is challenging. For example, in the past, thoracic epidurals have been widely used for pain control in thoracic surgery. However, it can limit mobilization and require a Foley catheter for monitoring urine output during surgery. Therefore, to achieve the highest possible compliance with the guidelines, a multidisciplinary approach facilitated by the surgical team is important. Several meta-analyses and systematic reviews have reported that ERAS is associated with a shorter length of hospital stays in patients who undergo lung resections [10, 11]. A previous study evaluated patients who were randomized into fast-track surgery (limited thoracotomy with early chest drain removal and intravenous anesthesia) or traditional surgery. Results showed that the ERAS fast-track approach was associated with a lesser postoperative pain, and lower incidence of complications, and hospitalization costs [12]. Moreover, another study revealed that the fast-track regimen was significantly effective in reducing postoperative pain and pulmonary complications [13]. However, Brunelli et al. found that ERAS was not beneficial in terms of cardiopulmonary complications, mortality, length of hospital stay, and readmission [3]. Our study did not identify differences in terms of complications or mortality between the ERAS and non-ERAS groups.

The ERAS programs were primarily introduced in several countries due to hospital costs. Previous studies have shown that the ERAS programs can reduce health care costs by decreasing hospitalization cost [14–17]. A systemic review and meta-analysis has reported that ERAS can promote postoperative recovery, thereby reducing hospitalization costs [5]. However, another report showed that ERAS was correlated with higher overall hospitalization costs due to prolonged preoperative hospitalization [4]. Since data on hospitalization costs for our study is unavailable, the effect of the ERAS program on hospitalization costs remains uncertain.

The use of VATS is a key aspect of ERAS. VATS can reduce the length of hospital stay and pulmonary complications after surgery [18]. Therefore, it is an ideal surgical method for ERAS. We had originally planned a subgroup analysis between VATS and OT based on the ERAS protocol. However, the numbers of cases in each group were an issue. Hence, we analyzed the interaction between ERAS protocol and surgical approaches. Results showed that ERAS was beneficial in VATS and OT. Patients who underwent OT based on ERAS had a shorter chest tube duration and length of hospital stay than those who underwent OT under the pre-ERAS protocol. Therefore, ERAS appears to be beneficial regardless of surgical approaches, but further studies with a larger sample size are needed to confirm these results.

## Limitations

Our study had several limitations. It was retrospective in nature and was conducted at a single institution. Therefore, there might be some selection biases and potential unmeasured confounding variables. Patients were selected for VATS or OT based on the surgeons’ preference; thus, selection bias could have existed. In the last few years, VATS surgery under ERAS has been introduced in our unit. Although we attempted to reduce the selection bias via propensity score analysis matching, there was still an imbalance in surgical approach between the two groups. Multivariable regression analysis with adjusted confounding factors that might affect interesting outcomes was performed to evaluate the interaction between the ERAS protocol and surgical approaches. Results showed that ERAS was beneficial. We believe that some types of bias may not be corrected via statistical analysis, and all the benefits derived in this study may be attributable to VATS. This is probably still true despite propensity matching. To assess the exact benefit of ERAS, further studies, including a single group of patients undergoing thoracotomy or all VATS, are warranted. Another limitation is the small sample size and short follow-up duration. Thus, a large national registry or randomized control trial must be conducted to further validate the benefit of ERAS in thoracic surgery. All costs including direct and indirect medical and non-medical costs are also the important issues and should be further investigated.

## Conclusions

The use of ERAS guidelines in pulmonary resection is beneficial. Although no significant differences were observed in postoperative complication rate and intensive care unit (ICU) stay between the two groups, patients in the post-ERAS group had a shorter postoperative chest tube duration, shorter hospital stays, shorter operative time, lower postoperative pain score, and lower volume of intraoperative blood loss. The perceived benefit of ERAS in our study was most likely driven by inclusion of VATS. Future studies should recruit only patients undergoing VATS to evaluate the independent effect of ERAS. Furthermore, the cost factor remains an issue in developing countries and should be explored more thoroughly for cost-effectiveness.

## Data Availability

The data that support the findings of this study are not openly available due to reasons of sensitivity and are available from the corresponding author upon reasonable request. Data are located in controlled access data storage at Personal computer of corresponding author.
